# Improvements in cognitive function and quantitative sleep electroencephalogram in obstructive sleep apnea after six months of continuous positive airway pressure treatment

**DOI:** 10.1093/sleep/zsac013

**Published:** 2022-01-13

**Authors:** Angela L D’Rozario, Camilla M Hoyos, Keith K H Wong, Gunnar Unger, Jong Won Kim, Andrew Vakulin, Chien-Hui Kao, Sharon L Naismith, Delwyn J Bartlett, Ronald R Grunstein

**Affiliations:** Faculty of Science, School of Psychology, University of Sydney, Sydney, New South Wales, Australia; Sleep and Circadian Research Group, Woolcock Institute of Medical Research, University of Sydney, Glebe, New South Wales, Australia; Healthy Brain Ageing Program, Brain and Mind Centre, University of Sydney, Sydney, New South Wales, Australia; Charles Perkins Centre, University of Sydney, Sydney, New South Wales, Australia; Faculty of Science, School of Psychology, University of Sydney, Sydney, New South Wales, Australia; Sleep and Circadian Research Group, Woolcock Institute of Medical Research, University of Sydney, Glebe, New South Wales, Australia; Healthy Brain Ageing Program, Brain and Mind Centre, University of Sydney, Sydney, New South Wales, Australia; Charles Perkins Centre, University of Sydney, Sydney, New South Wales, Australia; Sleep and Circadian Research Group, Woolcock Institute of Medical Research, University of Sydney, Glebe, New South Wales, Australia; Sydney Medical School, Faculty of Medicine and Health, University of Sydney, Sydney, New South Wales, Australia; Department of Respiratory and Sleep Medicine, Royal Prince Alfred Hospital, Camperdown, New South Wales, Australia; Sleep and Circadian Research Group, Woolcock Institute of Medical Research, University of Sydney, Glebe, New South Wales, Australia; Sleep and Circadian Research Group, Woolcock Institute of Medical Research, University of Sydney, Glebe, New South Wales, Australia; Department of Healthcare IT, Inje University, Inje-ro 197, Kimhae, Kyunsangnam-do, 50834,South Korea; Adelaide Institute for Sleep Health/FHMRI Sleep Health, College of Medicine and Public Health, Flinders University, Bedford Park, South Australia, Australia; Healthy Brain Ageing Program, Brain and Mind Centre, University of Sydney, Sydney, New South Wales, Australia; Faculty of Science, School of Psychology, University of Sydney, Sydney, New South Wales, Australia; Healthy Brain Ageing Program, Brain and Mind Centre, University of Sydney, Sydney, New South Wales, Australia; Charles Perkins Centre, University of Sydney, Sydney, New South Wales, Australia; Sleep and Circadian Research Group, Woolcock Institute of Medical Research, University of Sydney, Glebe, New South Wales, Australia; Sydney Medical School, Faculty of Medicine and Health, University of Sydney, Sydney, New South Wales, Australia; Sleep and Circadian Research Group, Woolcock Institute of Medical Research, University of Sydney, Glebe, New South Wales, Australia; Sydney Medical School, Faculty of Medicine and Health, University of Sydney, Sydney, New South Wales, Australia; Department of Respiratory and Sleep Medicine, Royal Prince Alfred Hospital, Camperdown, New South Wales, Australia

**Keywords:** power spectral analysis, performance, sleep-disordered breathing, positive airway pressure, neurophysiology

## Abstract

**Study Objectives:**

Untreated obstructive sleep apnea (OSA) is associated with cognitive deficits and altered brain electrophysiology. We evaluated the effect of continuous positive airway pressure (CPAP) treatment on quantitative sleep electroencephalogram (EEG) measures and cognitive function.

**Methods:**

We studied 167 patients with OSA (age 50 ± 13, AHI 35.0 ± 26.8) before and after 6 months of CPAP. Cognitive tests assessed working memory, sustained attention, visuospatial scanning, and executive function. All participants underwent overnight polysomnography at baseline and after CPAP. Power spectral analysis was performed on EEG data (C3-M2) in a sub-set of 90 participants. Relative delta EEG power and sigma power in NREM and EEG slowing in REM were calculated. Spindle densities (events/min) in N2 were also derived using automated spindle event detection. All outcomes were analysed as change from baseline.

**Results:**

Cognitive function across all cognitive domains improved after six months of CPAP. In our sub-set, increased relative delta power (*p* < .0001) and reduced sigma power (*p* = .001) during NREM were observed after the 6-month treatment period. Overall, fast and slow sleep spindle densities during N2 were increased after treatment.

**Conclusions:**

Cognitive performance was improved and sleep EEG features were enhanced when assessing the effects of CPAP. These findings suggest the reversibility of cognitive deficits and altered brain electrophysiology observed in untreated OSA following six months of treatment.

**Statement of Significance**

Altered sleep neurophysiology and impaired cognitive performance are common features of untreated obstructive sleep apnea. Our study examined the effects of 6 months of CPAP therapy on quantitative sleep EEG measures and cognitive functions known to be affected in sleep apnea. Increased delta EEG power and sleep spindle density during NREM sleep and improved cognition, mood, and daytime sleepiness were observed after CPAP. This suggests that CPAP can ameliorate sleep EEG abnormalities and cognitive deficits in OSA after 6 months of treatment. Further research is required to confirm whether this effect translates to improved cognitive trajectories over the longer term.

## Introduction

The effects of untreated obstructive sleep apnea (OSA) on daytime function in individuals are variable. Some individuals are symptomatic and report excessive daytime sleepiness and cognitive dysfunction, while others appear relatively unaffected and function well [1–3]. Impairments are commonly seen on cognitive tests of vigilance, attention, visuospatial abilities, executive functions, and some components of memory [4]. Reversal of these impairments with continuous positive airway pressure (CPAP) therapy, the “gold standard” treatment for OSA, is variable within individuals, with only limited or partial reversibility of cognitive deficits observed [5–8].

The daytime consequences of untreated OSA include increased risk for motor vehicle crashes and workplace accidents [9, 10]. Untreated OSA has also been implicated as a significant risk factor for developing dementia and mild cognitive impairment (MCI), an “at risk” stage for dementia [11]. Traditional polysomnography (PSG)-derived measures of OSA disease severity are inconsistently or weakly related to cognitive function [12, 13]. Therefore, it is important to clarify the recovery of cognitive outcomes that occur with OSA treatment, and to identify more sensitive markers that track cognitive function in this population.

Quantitative electroencephalography (EEG) measures of sleep microarchitecture provide sensitive markers of cognitive decline in ageing and neurodegeneration [14, 15] and cognitive deficits in patients with OSA [16, 17]. Case-controlled OSA studies demonstrate EEG abnormalities during NREM sleep including reduced slow wave frequency brain activity and sleep spindle deficits [18]. These EEG oscillations play a key role in sleep-mediated learning and memory processes, and abnormalities may underlie cognitive impairment in OSA. REM sleep EEG abnormalities are also observed in untreated OSA, with greater EEG slowing compared to controls [19]. Although there is a paucity of studies examining the recovery of sleep microarchitecture in OSA after CPAP, prior research in small samples (*n* ≤ 20) suggests that therapy may partially reverse some abnormalities in sleep EEG activity [20–23]. No known studies have examined sleep EEG measures at baseline (untreated) and associated changes in cognitive function after CPAP therapy.

In this study, we assessed the effects of 6 months of CPAP therapy on (1) cognitive performance across domains known to be impaired in OSA and (2) sleep EEG microarchitecture measures in NREM and REM sleep.

## Methods

### Participants

This study examined data collected from participants who were involved in a previously reported clinical trial [24]. Participants have been described in detail previously. In brief, eligible participants had newly diagnosed PSG-defined at least mild OSA (as defined as an apnea hypopnea index [AHI] > 5). Exclusion criteria included any previous or current use of CPAP and non-fluency in both written and spoken English. The Sydney South West Area Health Service Human Research Ethics Committee approved the protocol and all participants provided written informed consent. The study was registered with the Australia and New Zealand clinical trials registry (ANZCTR12606000065594).

### Study design and CPAP treatment

The original study examined the effect of a behavioural intervention on CPAP adherence over 6 months compared to a conservative intervention. This current paper examines the effect of CPAP treatment on sleep and cognitive function regardless of the behavioural intervention allocation. All participants underwent a standard in-laboratory manual CPAP pressure determination study and were provided a REMStar Pro (Respironics Inc Murrysville, PA) or a Sandman GoodKnight 420 Series Auto HH (Covidien, Mansfield, MA) for the duration of the study. CPAP usage data was measured objectively within the devices using a data card or memory key which was downloaded at each visit.

### Performance tasks

The test battery has been previously described [25]. In brief, the test battery comprised computerised cognitive tasks modelled on conventional neuropsychological tests [26] and the hand-held 10-min psychomotor vigilance task (PVT) [27]. The full test battery took approximately 30 min for participants to complete. During each visit, the battery was performed at the same time of day and on the same day of the attended sleep study. As part of the protocol, participants were instructed not to consume any stimulants such as caffeine on the testing days in the sleep laboratory.

The tasks undertaken during the computerised battery were the: (1) letter cancellation task which assessed attention, concentration, and visuospatial scanning ability or visuospatial neglect, plus measuring accuracy of selective attention; (2) Stroop text and Stroop colour assessed the inhibition of dominant responses, reflecting “higher-order” executive functions; (3) n-Back assessed working memory, encompassing short term memory storage, and information processing reflecting “central executive” processes (using the 2 and 3 back); and 4) The PVT hand-held task assessed sustained attention sensitive to sleep loss [28], identifying attentional lapses even in mild OSA [29].

### Self-report questionnaires

Participants completed questionnaires at baseline and 6 months for the assessment of sleepiness (Epworth Sleepiness Scale) [30], sleep-related quality of life (Functional Outcomes Sleep Questionnaire) [31] and symptoms of depression, anxiety, and stress (Depression Anxiety Stress Scale) [32].

### Polysomnography

In-laboratory PSG with an 8-hour time in bed opportunity (10 pm to 6 am) was performed at baseline (untreated) and after 6 months of CPAP treatment (patient’s usual machine in situ). PSGs were conducted across three sleep laboratories in Sydney, Australia: Woolcock Institute of Medical Research, Royal Prince Alfred Hospital, and Royal North Shore Hospital. Compumedics (Melbourne, Australia), Embla Titanium (Natus, CA, USA), or Alice-4 (Philips Respironics, Murrysville, PA, USA) PSG systems were used. A standardised PSG montage was used with EEG at scalp locations C3, C4, O1, O2 referenced to the contralateral mastoids at M1 and M2. Sleep staging and scoring were performed by registered sleep technologists [33, 34]. EEG was sampled at 200 Hz for Alice and 128 Hz for Compumedics and Embla Titanium. PSG recordings were exported into standardised digital European Data Format (EDF) prior to all subsequent quantitative EEG analyses.

### Quantitative analysis of sleep EEG

#### Power spectral analysis.

All night PSG recordings were subjected to automated EEG artefact processing using previously validated artefact detection threshold parameters [35]. Artefact-free epochs were analysed using a standard fast Fourier transform (FFT) with a rectangular weighted window for each non-overlapping 5-second epoch of EEG for central (C3-M2, C4-M1) channels. For quantitative analysis, data was derived from the C3-M2 derivation unless it was poor quality, in which case C4-M1 was used (14 of 180 EEG recordings). The FFT routine used requires the total number of data points in each epoch to be in the form of a power of 2, i.e. 128 (2^7^) or 256 (2^8^) data points. For EEG data sampled at a rate of 200 Hz, i.e. 1000 data points, the FFT was performed twice, to 512 data points selected from the beginning and end of each epoch. The resulting mean value for the epoch gives a greater weight to the middle-data points. We calculated absolute spectral power (µV^2^) in the delta, theta, alpha, sigma, and beta bands defined as EEG activity in each of the respective frequency ranges 0.5–4.5, 4.5–8, 8–12, 12–15, and 15–32 Hz. The EEG power for each sleep-staged 30-s epoch of the PSG recording was calculated by averaging data from up to 6 artefact-free 5-s epochs of EEG that comprised that 30-s recording segment. The weighted-average spectral power within the defined frequency bands was then computed for NREM (N2 and N3) and REM sleep stages. Relative power was our primary EEG spectral power measure. Relative power was calculated by dividing the absolute spectral power in a given frequency range e.g. delta power (0.5–4.5 Hz), by total power (0.5–32 Hz). Absolute power was also reported for completeness. To derive an index of EEG slowing during sleep we calculated the EEG Slowing Ratio during REM sleep i.e. a ratio of slow frequencies to fast frequencies (delta + theta)/ (alpha + sigma + beta).

#### Sleep spindle detection algorithm.

Spindle identification used an automatic sleep spindle detection algorithm, see Supplementary Material for detail. A band-passing Finite-Impulse-Response filter (11–16 Hz) was applied to the raw EEG signal, yielding a time course of EEG activity in the sigma frequency range. A Hilbert transformation was applied to extract envelopes of the sigma EEG activity using a threshold calculated independently for the C3-M2 and C4-M1 derivations. The threshold value for each channel was given by the formula: median amplitude (µV) + 1.0 × standard deviation amplitude of the signal. The duration threshold for spindle events was 0.5–3.0 s. An index of sleep spindle events per minute of N2 was calculated for overall spindle density (11–16 Hz, events per min), slow spindle density (11 ≤ fz ≤ 13 Hz; fz: frequency), and fast spindle density (13 < fz ≤ 16 Hz).

### Sham CPAP historical control data

To create an historical control dataset, we combined the data from 28 participants who completed two randomised, CPAP sham-controlled studies [36, 37]. Study A (ANZCTRN12608000301369) was a 12-week parallel group study in moderate-severe OSA. Study B (ANZCTRN12610000144011) was a factorial design testing the effect of 12 weeks of both therapeutic CPAP vs. sham CPAP and vardenafil vs. oral placebo in men with both moderate-severe OSA and erectile dysfunction. 28 participants who received sham CPAP were included in the analysis (15 and 13 in studies A and B, respectively).

### Statistical analysis

Analyses were performed using SAS V9.2 (SAS Institute). Data were considered statistically significant at *p* < .05 (two-sided) and are presented as mean (SD) or mean difference from baseline (95% CI), as indicated. Paired t tests determined the change from baseline after 6 months of CPAP for PSG, performance tasks, and quantitative EEG measures. Of primary interest were relative delta and sigma (spindle frequency activity) power in NREM, spindle density (overall) in N2, and the EEG slowing ratio in REM sleep. In an additional analysis, the changes were adjusted for the change in depression (DASS-D) at 6 months for the performance tasks. In a post hoc analysis, the same methods were used for the control group as for the CPAP group with the prespecified outcomes of NREM delta and sigma power, spindle density in N2, and the EEG slowing ratio in REM. Exploratory analyses determined the association between CPAP usage (hours/night) and the change in quantitative EEG measures, as well as the change in selected quantitative EEG measures and change in cognitive outcomes at 6 months using Pearson's correlation coefficients.

## Results

### Participant flow and characteristics

As previously described [24], 294 participants were screened, 206 participants were randomised into the intervention study, and 177 completed the 6-month follow-up visit. Of these, 167 had cognitive performance and questionnaire data at baseline and 6 months and are currently analysed. One hundred and thirty participants had PSG data at baseline and 6 months available for quantitative EEG analysis. EEG data from 40 participants were excluded due to excessive EEG artefact with a final sub-set of 90 participants with quantitative EEG data before and after treatment.

Baseline characteristics for the 167 participants with performance/questionnaire data, and 90 participants with quantitative sleep EEG data at baseline and 6 months are shown in Table 1. Average CPAP use (hours/night) over the 6 months was 4.3 (SD: 2.5, range: 0–10.1) and 4.5 (SD: 2.4, range: 0.05–10.1) hours per night in the group of 167 and 90 participants, respectively with approximately 60% of participants using their device 4 hours or more per night. There were no significant changes observed in BMI before and after treatment (mean change 0.0789 kg/m^2^; 95% CI −0.1339% to 0.2917%, *p* = 0.47).

**Table 1.** Baseline participant characteristics

	All participants (*n* = 167)	Sub-set with qEEG (*n* = 90)
Age (years)	49.7 (12.6)	50.6 (12.1)
Males, *n* (%)	125 (75%)	65 (72%)
Body Mass Index (kg/m^2^)	33.5 (7.6)	32.6 (6.7)

Data are mean (standard deviation) or count (%) unless otherwise stated. qEEG: quantitative electroencephalography.

### Cognitive tasks and self-report questionnaires

Six months of CPAP treatment significantly improved visuospatial spanning (average hits and final duration on LCT), the ability to inhibit cognitive interference as assessed by the Stroop task, working memory (percent accuracy on both 2 and 3 n-back), and sustained attention (mean reaction time, mean reciprocal reaction time and mean reciprocal of slowest 10% reaction time on PVT), Table 2. After adjusting for the change in depression after 6 months (DASS-D), all improvements remained except for Stroop text accuracy and reaction time (data not shown).

**Table 2.** Cognitive performance and sleepiness and mood questionnaires before and after 6 months of CPAP treatment

	Baseline	CPAP	Difference	Effect size	*P*
Sleepiness and mood					
Epworth sleepiness scale_(lower better) (*n* = 167)_	11.97 (4.90)	7.67 (4.61)	−4.30 (−5.05 to −3.55)	0.88	**<.0001**
DASS-depression_(*n* = 162)_	11.10 (9.48)	7.67 (9.68)	−3.43 (−4.69 to −2.17)	0.36	**<.0001**
DASS-anxiety_(*n* = 163)_	9.62 (8.18)	6.90 (7.76)	−2.72 (−3.95 to −1.50)	0.33	**<.0001**
DASS-stress_(*n* = 163)_	13.93 (9.06)	9.96 (8.34)	−3.96 (−5.21 to −2.72)	0.44	**<.0001**
FOSQ total score_(*n* = 148)_	14.78 (3.35)	16.99 (2.92)	2.21 (1.73 to 2.70)	0.66	**<.0001**
Visuospatial scanning (letter cancellation task)					
Average hits_(higher better) (n = 162)_	50.76 (13.00)	55.11 (12.24)	4.34 (3.28 to 5.40)	0.33	**<.0001**
Average omissions_(lower better) (n = 162)_	3.68 (5.23)	3.31 (5.35)	−0.37 (−1.15 to 0.41)	0.07	.35
Average commissions_(lower better) (*n* = 162)_	0.97 (1.60)	0.80 (1.56)	−0.17 (−0.43 to 0.1)	0.11	.21
Hits final trial_(higher better)* (*n* = 159)_	297.00 (17.0)	297.00 (14.0)	0.00 (9.00)	0.00	.93
Omissions final trial_(lower better)* (*n* = 159)_	13.00 (17.00)	13.00 (14.00)	0.00 (9.00)	0.00	.93
Commissions final trial_ (lower better) (*n* = 159)_	4.15 (10.11)	3.12 (4.86)	−1.03 (−2.46 to 0.40)	0.10	.16
Duration final trial (s)_(lower better) (*n* = 159)_	367.66 (164.83)	320.12 (85.65)	−47.55 (−70.23 to −24.86)	0.29	**<.0001**
Executive function (Stroop)					
Text accuracy (%)_(higher better) % (*n* = 161)_	92.85 (17.39)	96.02 (10.93)	3.17 (0.02 to 6.31)	0.18	**.049**
Text RT (sec)_(lower better) (*n* = 161)_	1.28 (0.47)	1.21 (0.35)	−0.08 (−0.14 to −0.01)	0.17	**.02**
Colour accuracy (%)_(higher better) (*n* = 160)_	79.68 (25.78)	89.52 (21.92)	9.84 (5.44 to 14.23)	0.38	**<.0001**
Colour RT (s)_(lower better) (*n* = 160)_	1.44 (0.58)	1.28 (0.47)	−0.16 (−0.24 to −0.08)	0.28	**<.0001**
Working memory (n-back)					
2-Back accuracy NTGA (%)_(higher better) (*n* = 162)_	0.65 (0.26)	0.73 (0.26)	0.08 (0.04 to 0.12)	0.31	**<.0001**
3-Back accuracy NTGA (%)_(higher better) (*n* = 161)_	0.56 (0.24)	0.65 (0.24)	0.09 (0.06 to 0.13)	0.38	**<.0001**
Sustained attention (psychomotor vigilance task)					
Mean RT_(lower better)_	300.84 (76.98)	286.15 (72.36)	−14.69 (−28.65 to −0.73)	0.19	**.04**
Mean reciprocal RT_(higher better)_	3.78 (0.56)	3.90 (0.54)	0.12 (0.03 to 0.21)	0.21	**.009**
Mean fastest 10% RT_(lower better)_	203.25 (26.08)	200.19 (22.24)	−3.06 (−6.74 to 0.63)	0.12	.10
Mean slowest 10% reciprocal RT_(higher better)_	2.23 (0.64)	2.50 (0.65)	0.27 (0.14 to 0.40)	0.42	**<.0001**
Lapses_(lower better)_	4.30 (6.54)	2.51 (4.42)	−1.80 (−2.89 to −0.70)	0.28	**.002**

Data are mean (standard deviation) and mean difference (95% CI) or median (IQR) and median difference (IQR) as indicated by a *. *P* values are calculated using paired t tests. DASS: Depression, Anxiety and Stress Scale; FOSQ: Functional Outcomes of Sleep Questionnaires; RT: reaction time. Bold values indicate *p* < 0.05.

Additionally, there were improvements in subjective sleepiness (ESS), depressive, anxiety, and stress symptoms (DASS), and quality of life relating to sleep (total FOSQ score), all *p* < 0.0001, Table 2.

### Sleep architecture

Sleep architecture and respiratory indices before and after the treatment period are presented in Table 3. Stage N2 sleep significantly decreased whereas slow wave sleep (N3), REM sleep, and sleep efficiency increased with 6 months of CPAP treatment. CPAP improved all OSA parameters: arousal index, AHI, 3% oxygen desaturation index, and minimum oxygen saturation during sleep.

**Table 3.** Polysomnography measures before and after 6 months of CPAP treatment

	Baseline	CPAP	Difference	*P*
Time in bed (min)	460.44 (39.97)	450.36 (29.15)	−10.08 (−19.93 to −0.24)	**.0448**
Total sleep time (min)	367.92 (54.19)	386.79 (43.39)	18.87 (7.59 to 30.15)	**.0013**
NREM sleep (min)	287.59 (52.54)	286.79 (39.87)	−0.81 (−12.35 to 10.73)	.89
Stage N1 (min)	19.39 (17.97)	18.53 (14.20)	−0.86 (−4.91 to 3.20)	.68
Stage N2 (min)	232.23 (55.80)	172.63 (56.87)	−59.60 (−76.04 to −43.16)	**<.0001**
Stage N3,SWS (min)	55.36 (39.56)	114.16 (51.27)	58.79 (46.14 to 71.45)	**<.0001**
REM sleep (min)	60.94 (31.36)	81.47 (25.95)	20.53 (13.19 to 27.87)	**<.0001**
Sleep efficiency (%)	80.15 (11.15)	86.09 (9.58)	5.95 (3.64 to 8.25)	**<.0001**
Wake after sleep onset (min)	68.49 (44.31)	49.80 (37.83)	−18.67 (−29.05 to −8.29)	**.0006**
Arousal index (events/h)	34.06 (21.05)	17.92 (9.48)	−16.14 (−21.22 to −11.06)	**<.0001**
AHI (events/h)	34.80 (24.74)	4.33 (3.41)	−30.47 (−35.89 to −25.06)	**<.0001**
ODI (events/h)	30.23 (26.81)	3.68 (3.38)	−26.55 (−33.06 to −20.05)	**<.0001**
Minimum SaO_2_ (%)	78.19 (11.28)	90.58 (3.61)	12.39 (9.64 to 15.13)	**<.0001**

Data are mean (standard deviation) and mean difference (95% CI). *P* values are calculated using paired t tests. AHI: Apnea hypopnea index; ODI: 3% oxygen desaturation index; NREM: Non rapid eye movement; REM: rapid eye movement; SaO_2_: oxygen saturation; SWS: slow wave sleep. Bold values indicate *p* < 0.05.

### Quantitative sleep EEG

#### NREM sleep spectral power.

**Figure 1.**
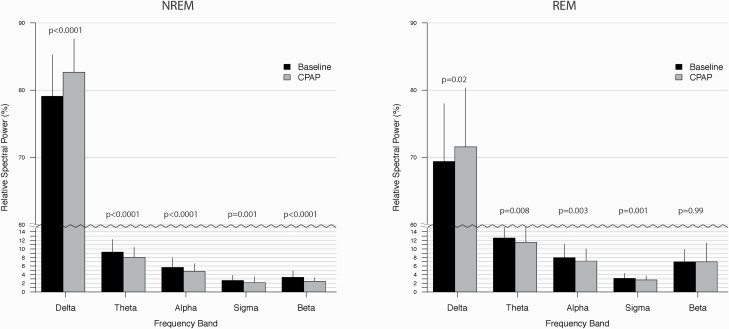
Relative EEG spectral power during NREM and REM sleep on the baseline (black bars) and CPAP treatment (grey bars) nights.

CPAP treatment increased relative delta power and reduced relative sigma power during NREM sleep (Figure 1, Supplementary Table S1). Relative EEG power was also reduced in theta, alpha, beta frequency ranges.

CPAP increased absolute delta and sigma power during NREM sleep relative to baseline as well as absolute EEG power in the theta and alpha frequency ranges (Supplementary Figure S1 and Supplementary Table S2).

#### Sleep spindle events.

CPAP treatment significantly increased overall spindle density (11–16 Hz, events per min), slow spindle density (11–13 Hz), and fast spindle density (>13–16 Hz) during N2 sleep (Table 4).

**Table 4.** Spindle density during stage N2 sleep before and after 6 months of CPAP treatment

	Baseline	CPAP	Difference	*P*
Overall spindle density (events/min)	1.65 (0.88)	1.84 (0.97)	0.19 (0.07 to 0.32)	**.003**
Fast spindle density (events/min)	1.09 (0.73)	1.18 (0.83)	0.62 (0.40 to 0.84)	<.0001
Slow spindle density (events/min)	0.56 (0.54)	0.66 (0.54)	−0.43 (−0.60 to −0.25)	**<.0001**

*N* = 90. Data are mean (standard deviation) and mean difference (95% CI). *P* values are calculated using paired t tests. Bold values indicate *p* < 0.05. Bold values indicate *p* < 0.05.

#### EEG slowing in REM sleep.

The EEG slowing ratio during REM sleep increased after CPAP (EEG slowing ratio: Baseline vs. CPAP mean (SD) 5.21 (2.18) vs. 5.88 (2.90), change 0.67 [95% CI: 0.16% to 1.18%], *p* = .01).

#### REM sleep spectral power.

Relative delta power during REM sleep was increased after treatment while theta, alpha, and sigma power decreased with no significant change observed in beta power (Figure 1, Supplementary Table e1).

CPAP increased absolute EEG power across all frequency ranges in REM sleep (Supplementary Figure S1, Supplementary Table S2).

#### Associations between CPAP usage and change in sleep EEG microarchitecture and cognition.

Post hoc analysis showed greater CPAP usage (hours/night) was associated with a larger change in relative delta power (*r* = 0.205, *p* = .052) and change in relative sigma power (*r* = −0.215, *p* = .041) in NREM sleep. CPAP usage was not significantly related to any other spectral power or spindle measure in NREM or during REM sleep. Exploratory analysis which examined possible associations between CPAP usage and a priori variables selected from each cognitive test based on the largest change per and post CPAP (3-back accuracy, PVT slowest 10% reciprocal reaction time, Stroop accuracy, Letter cancellation task average hits) revealed a small but significant correlation with Stroop accuracy (*r *= 0.16, *p* = .03) but no other associations. Additional exploratory analysis showed no associations between change in the selected cognitive outcomes and change in relative delta power in NREM, relative sigma power in NREM, and N2 spindle density (data not shown).

### Sham CPAP: historical control comparison

Twenty-eight male participants were included in the historical sham CPAP group (mean age (SD) = 42.7 (11.8); AHI [events/h] = 44.5 (25.0); body mass index [kg/m^2^] = 32.5 (5.1); and ESS score = 10.8 (3.7)). Mean sham CPAP use across the 12 weeks period was 2.1 (1.9) hours per night. As expected, there were no changes in AHI after sham CPAP (mean difference = 1.54 [95% CI: −5.60% to 8.68%], *p* = .66) or any sleep stage durations. There were no changes after 12 weeks of sham CPAP treatment in any of the pre-specified outcomes: relative NREM delta power (baseline vs. sham CPAP, mean (SD) 78.51 (6.49) vs. 79.40 (5.89), mean difference [95% CI] 0.90 [: −1.15% to 2.94%], *p* = .38; stage N2 sleep overall spindle density: 2.56 (1.06) vs. 2.56 (1.07), −0.002 [−0.25 to 0.24], *p* = .98; and REM EEG slowing ratio: 4.96 (2.27) vs. 5.37 (1.89), −.41 [−1.53 to 0.70], *p* = .45).

## Discussion

This is the largest study to date evaluating changes in sleep EEG microarchitecture after CPAP therapy. After CPAP, we observed improvements in disease severity metrics and altered sleep architecture resulting in greater sleep efficiency, increased duration of SWS and REM sleep, and reduced time spent in stage N2 sleep. Sleep microarchitecture also changed with increased relative and absolute delta power in NREM and greater sleep spindle density in N2. Performance across all cognitive domains was enhanced and sleepiness and mood improved after CPAP in this large clinical group of treatment naïve patients.

### NREM sleep architecture

We observed increased slow frequency EEG activity (relative delta power) during NREM sleep after CPAP with accompanied decreases in relative power across the four other frequency ranges of theta, alpha, sigma, and beta. The enhancement of delta power was the most marked response suggesting a recovery in the depth of deep sleep after CPAP. This occurred despite greater EEG activation as shown by increased absolute power across all frequency ranges except in the faster frequency beta range (Supplementary Data).

We also observed significant increases in spindle density after CPAP suggesting a recovery of this hallmark feature of N2 sleep. Although the amount of NREM sleep was not significantly different before and after CPAP treatment, the duration of slow wave sleep (N3) increased while N2 sleep decreased, and supports previous findings [18].

Two other small studies have reported similar results where slow wave activity during NREM sleep increased after 6 months [18] and 9 months [21] of CPAP treatment. In the former study, 15 patients with moderate to severe OSA showed enhanced sleep depth (the proportion of NREM sleep containing EEG brain waves slower than 4.0 Hz) at frontal and central brain regions after 6 months of CPAP compared to baseline. In some regions, this was normalized to the levels observed in control participants [18].

Partial recovery of sleep spindles was observed in a smaller study of 20 patients with OSA following 6 months of CPAP with increased central spindle density after treatment but with some persistent spindle abnormalities observed [20]. Spindle counts in N2 were increased after only one night of CPAP suggesting an acute response [38]. Although the sample size of this study was large (*n* = 73), the spindle detection methodology was unclear and spindles were identified within a one hour period of N2 sleep. In the current study, we analysed all-night EEG data before and after CPAP treatment showing significant enhancement of slow wave activity and spindles during sleep.

### REM sleep architecture

The duration of REM sleep was also increased after CPAP. During REM sleep we observed increased absolute power in all frequency ranges, as well as increased relative delta power and EEG slowing ratio. Only one study has examined the recovery of REM sleep microarchitecture following treatment with CPAP but none have assessed EEG slowing in relation to cognitive recovery. Morisson et al showed a significant decrease in REM absolute delta power after 6–9 months of CPAP in 14 people with severe OSA but no change in other frequency ranges. In the same study, frontal and central EEG slowing ratio in REM sleep was lower after CPAP but this was not significant after multiple comparison corrections [22]. Possible explanations for this discrepancy include different samples studied and methodological differences (90-sec segment of REM EEG compared to all night artefact-free REM EEG). There is the possibility that the reversal of pathological EEG slowing previously observed in severe OSA after CPAP was masked by the heterogeneity of sleep apnea severity in our larger sample.

### Cognitive performance

After CPAP treatment, we observed improvements in working memory (n-back) and set-shifting (Stroop) which fall under the domain of executive function. Previous studies and meta-analyses have also found improvements in executive function after CPAP treatment [39–41]. Improvements in set-shifting were observed but not in working memory which may relate to shorter treatment duration of 1–12 weeks in prior studies [40, 41]. Executive function is impaired in OSA compared to individuals without OSA [25, 42] and our results show that improvement in these areas is possible with CPAP treatment. We observed better sustained attention with CPAP and found improved outcomes in PVT measures as per another study [43] which was not evident in a milder OSA group [41]. Our results are in contrast to the largest randomised sham-controlled study which showed no differences in neurocognitive outcomes at 6 months. This finding was posited to be due to a lack of impairment at baseline, but improvements were seen in the sub-group of severe patients [8]. Furthermore, they were clinically less symptomatic at baseline (2 points lower on the ESS than the current study) which could explain the lack of improvement as decreasing sleepiness improves attention [7], and plays an important role in the uptake of treatment.

### Strengths and limitations

There are a number of strengths and limitations of the current study. This is the largest and longest study investigating the effects of CPAP on EEG and the largest to report both EEG and cognitive outcomes in OSA. We acknowledge this is an uncontrolled open-label study. This reflects the current difficulties in obtaining ethical approval and recruitment targets for sham or no treatment controls in sleepy patients with OSA for periods as long as 6 months. The lack of an age and education-matched control group also did not allow us to determine whether these improvements normalised. However, we have focused on objective rather than subjective outcome measures and utilised a pre-existing sham-CPAP sample as an historical control group for sleep EEG data. This sample showed no change in primary EEG markers suggestive of a limited placebo effect but had limitations in being all male and with lower sham CPAP usage and shorter duration of treatment than in our patients on active CPAP. We also acknowledge there is night-to-night variability in polysomnography measures of sleep that may have contributed to the differences observed before and after CPAP. However, our recent analysis of EEG profiles on patients with OSA recorded in the sleep laboratory on two different nights showed remarkable intra-individual stability in NREM delta and sigma power [44]. We used a standardised computerised battery of performance tasks assessing cognitive domains known to be impaired in OSA [25] with demonstrated sensitivity to CPAP treatment. However, these are not clinical neuropsychological measures. Therefore, without normative data or a control group, we cannot ascertain how impaired the sample was. Additionally, we are unable to determine the pathways by which the improvement in cognitive function may have occurred, specifically the extent to which changes in sleep microarchitecture that were also evident after CPAP contributed to these effects. Further randomised controlled studies that are able to carefully manipulate sleep microarchitecture and measure the effects on cognitive function may help elucidate these pathways, though we recognise that conducting such studies in OSA populations is complex. We also acknowledge the potential limitations of adopting an automated algorithm to detect sleep spindle events. The performance of automated spindle detection algorithms is imperfect when compared to manually identified sleep spindles by trained sleep technologists [45]. However, the validation process undertaken to test the algorithm used in the current study showed comparable agreement metrics to other published algorithms. Finally, we did not assess the domain of episodic memory, which is known to be impaired in OSA [46] and may even be an early hallmark feature of dementia.

## Conclusions and Future Research

The current study demonstrated the reversibility of sleep EEG abnormalities and cognitive improvements in OSA after 6 months of CPAP treatment. It is currently unknown whether this effect translates to improved cognitive trajectories over the longer term.

Importantly, OSA has been implicated as a risk factor for the development of mild cognitive impairment and dementia [11], and CPAP may slow cognitive decline [47], but robust evidence is required. In healthy older people changes in quantitative sleep EEG measures that precede the clinical onset of cognitive decline [14] support the potential utility of EEG as a biomarker for dementia progression. Future longitudinal studies in OSA are required to explore this trajectory. Furthermore, studies combining multi-modal neuroimaging and high-density EEG will elucidate potential mechanisms and targeted treatment approaches.

## Supplementary Material

Supplementary material is available at *SLEEP* online.

**Figure S1.** Absolute EEG spectral power during NREM and REM sleep on the baseline (black bars) and CPAP treatment (grey bars) nights.

**Figure S2. Automatic algorithm spindle detection schematic.**

**Table S1.** Relative EEG spectral power during NREM and REM sleep before and after CPAP treatment.

**Table S2.** Absolute EEG spectral power during NREM and REM sleep before and after CPAP treatment.

zsac013_suppl_Supplementary_Material
