# Supplementary material for: Improvements in cognitive function and quantitative sleep electroencephalogram in obstructive sleep apnea after six months of continuous positive airway pressure treatment
Source: Sleep. 2022 Jan 13;45(6):zsac013. doi: 10.1093/sleep/zsac013 (PMC9189957; doi:10.1093/sleep/zsac013)
Supplement: zsac013_suppl_Supplementary_Material [file zsac013_suppl_supplementary_material.docx]

# Supplementary Material

**Title:** Improvements in Cognitive Function and Quantitative Sleep EEG in OSA after Six Months of CPAP Treatment

**Contributing authors and affiliations:**

Angela L. D’Rozario^1,2,3,4*^, Camilla M. Hoyos^1,2,3,4*^, Keith K.H. Wong^2,5,6^, Gunnar Unger^2^, Jong Won Kim^2,7^, Andrew Vakulin^8^, Chien-Hui Kao^3^, Sharon L. Naismith^1,3,4^, Delwyn J. Bartlett^2,5^, Ronald R. Grunstein^2,5,6^

*(authors contributed equally to the manuscript)

^1^The University of Sydney, Faculty of Science, School of Psychology, Sydney, New South Wales, Australia ^2^Woolcock Institute of Medical Research, University of Sydney, Glebe, New South Wales, Australia ^3^Healthy Brain Ageing Program, Brain and Mind Centre, University of Sydney, Sydney, New South Wales, Australia ^4^Charles Perkins Centre, University of Sydney, Sydney, New South Wales, Australia ^5^The University of Sydney, Faculty of Medicine and Health, Sydney, New South Wales, Australia ^6^Royal Prince Alfred Hospital, Camperdown, New South Wales, Australia ^7^Department of Healthcare IT, Inje University, Inje-ro 197, Kimhae, Kyunsangnam-do, 50834, South Korea ^8^Adelaide Institute for Sleep Health / FHMRI Sleep Health, College of Medicine and Public Health, Flinders University, Bedford Park, SA

**Correspondence to:** Dr Angela D’Rozario, Woolcock Institute of Medical Research, PO Box M77, Missenden Road, Sydney NSW 2050 +612 9114 0435 [angela.drozario@sydney.edu.au](mailto:angela.drozario@sydney.edu.au)

## **Figure S1** Absolute EEG spectral power during NREM and REM sleep on the baseline (black bars) and CPAP treatment (grey bars) nights

## **
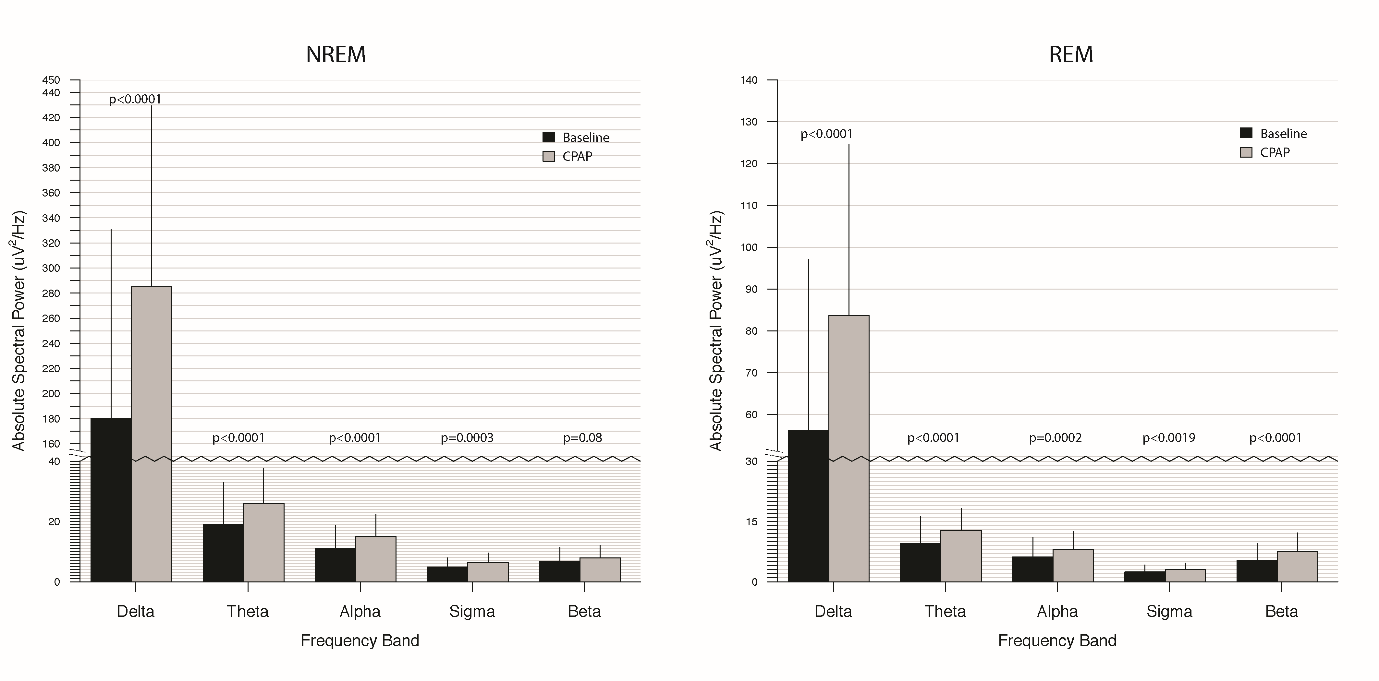
**

## **Table S1** Relative EEG spectral power during NREM and REM sleep before and after CPAP treatment.

|  | **Baseline** | **CPAP** | **Difference** | ***p*** |
| --- | --- | --- | --- | --- |
| **NREM Sleep** | | | | |
| Delta | 79.07 (6.17) | 82.64 (4.96) | 3.57 (2.31 to 4.82) | ***<0.0001*** |
| Theta | 9.27 (2.94) | 8.04 (2.34) | -1.22 (-1.74 to -0.71) | ***<0.0001*** |
| Sigma | 2.63 (1.22) | 2.13 (1.32) | -0.50 (-0.80 to 0.21) | ***0.001*** |
| Alpha | 5.66 (2.29) | 4.74 (1.79) | -0.92 (-1.30 to -0.54) | ***<0.0001*** |
| Beta | 3.37 (1.52) | 2.45 (0.90) | -0.93 (-1.26 to -0.59) | ***<0.0001*** |
| **REM Sleep** | | | | |
| Delta | 69.39 (8.59) | 71.56 (8.76) | 2.17 (0.40 to 3.94) | ***0.02*** |
| Theta | 12.53 (3.39) | 11.52 (3.58) | -1.00 (-1.73 to -0.27) | ***0.008*** |
| Sigma | 3.12 (1.13) | 2.76 (1.00) | -0.36 (-0.58 to -0.15) | ***0.001*** |
| Alpha | 7.98 (3.13) | 7.17 (2.86) | -0.80 (-1.32 to -0.29) | ***0.003*** |
| Beta | 6.98 (2.92) | 6.98 (4.40) | 0.001 (-0.83 to 0.83) | *0.99* |
| EEG Slowing ratio | 5.21 (2.18) | 5.88 (2.90) | 0.67 (0.16 to 1.18) | ***0.01*** |

N=90. Data are mean (standard deviation) and mean difference (95%CI). P values are calculated using paired t tests. REM: rapid eye movement; NREM: non-rapid eye movement; EEG, electroencephalography.

## **Table S2** Absolute EEG spectral power during NREM and REM sleep before and after CPAP treatment.

|  | **Baseline** | **CPAP** | **Difference** | ***p*** |
| --- | --- | --- | --- | --- |
| **NREM Sleep** |  |  |  |  |
| Delta | 179.87 (151.02) | 285.14 (144.12) | 105.27 (61.73 to 148.80) | ***<0.0001*** |
| Theta | 18.98 (13.99) | 25.96 (11.64) | 6.98 (4.07 to 9.88) | ***<0.0001*** |
| Sigma | 4.93 (3.01) | 6.42 (3.24) | 1.50 (0.70 to 2.29) | ***0.0003*** |
| Alpha | 11.09 (7.56) | 15.08 (7.38) | 4.00 (2.17 to 5.82) | ***<0.0001*** |
| Beta | 6.71 (4.81) | 7.89 (4.18) | 1.18 (-0.13 to 2.48) | *0.08* |
| **REM Sleep** |  |  |  |  |
| Delta | 56.11 (40.98) | 83.59 (40.89) | 27.49 (15.62 to 39.36) | ***<0.0001*** |
| Theta | 9.60 (6.60) | 12.74 (5.51) | 3.14 (1.73 to 4.55) | ***<0.0001*** |
| Sigma | 2.43 (1.76) | 3.06 (1.55) | 0.63 (0.24 to 1.03) | ***0.0019*** |
| Alpha | 6.19 (4.89) | 7.99 (4.56) | 1.81 (0.89 to 2.73) | ***0.0002*** |
| Beta | 5.37 (4.15) | 7.56 (4.63) | 2.19 (1.17 to 3.20) | ***<0.0001*** |

N=90. Data are mean (standard deviation) and mean difference (95%CI). P values are calculated using paired t tests. NREM: Non Rapid Eye Movement; REM: Rapid Eye Movement.

*Automated Spindle Detection Algorithm Validation*

Reference standard: Manual Spindle Identification

Sleep spindles (n=4426) from 200 epochs per study of stage N2 sleep were manually identified at the central EEG derivation referenced to the right mastoid electrode (C3-M2) in a sample of 20 all night in-laboratory polysomnography recordings (10 PSGs for training and 10 PSGs for validation) by two trained sleep technologists selected from untreated individuals with obstructive sleep apnea (OSA) and healthy young adults (HYA).

Studies were visually inspected in 30-second epochs using REMLogic software (Natus, San Carlos, CA, USA), by plotting both raw EEG (C3-M2) trace and a filtered trace (11-26.5Hz) on the display. The criteria for manual spindle identification involved: a clear, distinct spindle event observable in the raw trace; with frequency range of 11-16Hz; a duration of 0.3-3 seconds and comprising a diamond shape. In the case of partially overlapping spindles or a short inter-spindle distance, two separate spindles were scored if the sigma activities returned to baseline amplitude and duration criteria were satisfied. Reference standard spindle events were marked from onset to offset and exported with start time and duration details.

Automated Sleep Spindle Detection Algorithm

An automatic sleep spindle detection tool was developed and written in Java, version 1.6 (Oracle, Santa Clara, CA, USA). The tool’s algorithm computationally performs the following steps which are depicted in *Figure S2*. First, a 128 order band-passing Finite-Impulse-Response filter (11-16 Hz) is applied to the raw EEG signal, yielding a time course of sigma activity with duration threshold 0.5 ≤ duration ≤ 3.0 seconds. A Hilbert transformation is then applied to extract envelopes of the sigma activities. The spindles were identified according to the relative amplitude threshold, where the relative threshold value was calculated by the formula: median + α * standard deviation of the amplitude (µV).

## **Figure S2.** Automatic algorithm spindle detection schematic

##
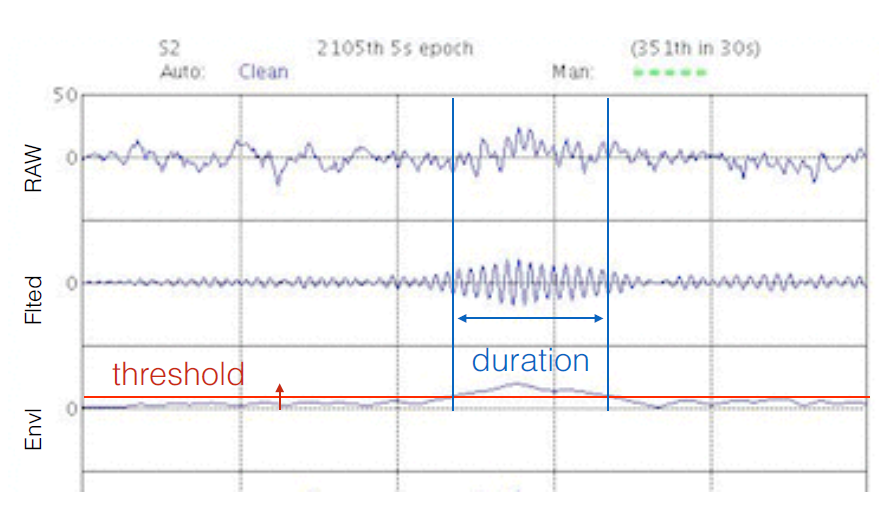
 Five second epoch of artefact free EEG. RAW, raw EEG signal; Flted, signal filtered for sigma activity; Envl, envelope of activity in the frequency range of sigma and meeting duration criteria.

Algorithm training

The training of the spindle detection algorithm included EEG data from 10 PSG recordings (5 OSA and 5 HYA) to identify spindles in 200 epochs of N2 sleep corresponding to the reference standard. The algorithm was trained using three amplitude threshold methods: when amplitudes were above either an absolute threshold (5 or 8 µV), or according to a relative threshold, where the relative threshold value was calculated by the formula: median + α * standard deviation of the amplitude (µV) and calculated independently for each EEG derivation.

For the relative amplitude method, the optimal α value was determined by comparing the performance of the algorithm at multiple α values in range 0.4-2.0, in increments of 0.1. In the case of partially overlapping spindles or a short inter-spindle distance, spindles were considered a single event when the inter-spindle amplitude was above threshold and duration criteria was satisfied.

Precision denotes the proportion of spindles detected by the algorithm correctly identified compared to the reference standard (manual detection), and was calculated by TP / (TP+FP) where true positives (TP) are spindles scored both manually and automatically and false positive (FP) spindles are scored automatically but not manually.

Recall denotes the proportion of manual spindle events correctly identified by the algorithm, calculated by TP / (TP+FN) where false negatives (FN) are spindles scored manually but not by the algorithm.

Performance accuracy was assessed by a cost function calculated using the formula [square root ((1 – Precision) * (1 – Precision) + (1 – Recall) * (1 - Recall))].

Best performance was achieved using the relative threshold methods with α= 1.0.

Algorithm validation

The sleep spindle algorithm, using a relative amplitude threshold (α = 1.0) was applied to the validation set of 10 PSG recordings (5 OSA and 5 HYA) to identify spindles in 200 epochs of N2 sleep corresponding to the reference standard. Performance of the automatic algorithm against the reference standard was assessed using Java scripts written specifically to calculate the precision, recall and cost of each amplitude threshold value.

Precision and recall performance of the algorithm for the OSA group was 0.87 and 0.52 respectively and from the healthy group was 0.79 and 0.75. Overall, precision for the 10 PSGs was 0.83 and recall was 0.64. Validation results are displayed in Table S3.

## **Table S3**. Precision and Recall results for the training and validation data sets

## Algorithm training performance (precision and recall defined in text) for three threshold methods: relative amplitude, absolute amplitude at 5 and 8 microvolts (µV). Validation performance in OSA (obstructive sleep apnea) and HYA (healthy younger adults) at relative amplitude threshold.

|  |  |  | **Amplitude Thresholds** | | | | | |
| --- | --- | --- | --- | --- | --- | --- | --- | --- |
|  |  |  | **Relative (α = 1)** | | **Absolute 5µV** | | **Absolute 8 µV** | |
|  |  | | **Precision** | **Recall** | **Precision** | **Recall** | **Precision** | **Recall** |
| **Training** | **OSA**  **(n=5)** | | 0.77 ± 0.07 | 0.65 ± 0.07 | 0.78 ± 0.17 | 0.62 ± 0.23 | 0.75 ± 0.42 | 0.20 ± 0.27 |
|  | **HYA**  **(n=5)** | | 0.73 ± 0.13 | 0.75 ± 0.16 | 0.68 ± 0.14 | 0.81 ± 0.07 | 0.87 ± 0.06 | 0.33 ± 0.21 |
|  | **All**  **(n=10)** | | **0.75 ± 0.10** | **0.70 ± 0.13** | **0.73 ± 0.16** | **0.72 ± 0.19** | **0.81 ± 0.29** | **0.27 ± 0.24** |
| **Validation** | **OSA**  **(n=5)** | | 0.87 ± 0.04 | 0.52 ± 0.15 | - | - | - | - |
|  | **HYA**  **(n=5)** | | 0.79 ± 0.05 | 0.75 ± 0.07 | - | - | - | - |
|  | **All**  **(n=10)** | | **0.83 ± 0.06** | **0.64 ± 0.16** | - | - | - | - |

Algorithm Performance

For comparison purposes, *Table S4* shows the F_1_-scores for the automated spindle detection algorithm used in the current study and other published algorithms. Accuracy was evaluated using an F_1_-score to compare automated and manual (gold standard reference) methods of spindle detection. F-1 scores range between 1 and 0, where 1 is equivalent to perfect precision and recall. Finally, the F_1_-score generated from our automated spindle detection algorithm was compared to six published algorithms that were previously evaluated.^1^ The optimal α amplitude threshold value of 1.0 was chosen based on the prior validation of the algorithm, and based on this threshold the F_1_-scores of the algorithm were 0.77 for the Obstructive Sleep Apnea (age: mean 44.6 ± 10.0 years) and Healthy Younger Adults (age 27.8 ± 2.4 years) groups. When compared to the previously published algorithms, our algorithm demonstrated higher F_1_-scores.

## **Table S4.** F1-scores for Algorithm

| **Performance of different spindle detection algorithms** | | **F1-scores** |
| --- | --- | --- |
| *Automated Spindle Detection Algorithm used in current study* | | |
|  | Obstructive Sleep Apnea | Mean ± SD 0.77 ± 0.07 |
|  | Healthy Younger Adults | 0.77 ± 0.06 |
| *Spindle Detection Algorithms detailed by Warby et al. 2014^1^* | | |
|  | Algorithm 1 | 0.28 |
|  | Algorithm 2 | 0.28 |
|  | Algorithm 3 | 0.21 |
|  | Algorithm 4 | 0.50 |
|  | Algorithm 5 | 0.52 |
|  | Algorithm 6 | 0.41 |
| *Algorithm 1-6 are from Warby et al. 2014.^1^* | | |

**References**

1. Warby SC, Wendt SL, Welinder P, et al. Sleep-spindle detection: Crowdsourcing and evaluating performance of experts, non-experts and automated methods. Nature Methods. 2014; 11 (4): 385-392.
